# Resveratrol Improves Hyperuricemia and Ameliorates Renal Injury by Modulating the Gut Microbiota

**DOI:** 10.3390/nu16071086

**Published:** 2024-04-07

**Authors:** Yuqing Zhou, Yupeng Zeng, Ruijie Wang, Juan Pang, Xin Wang, Zhijun Pan, Yufeng Jin, Yu Chen, Yan Yang, Wenhua Ling

**Affiliations:** 1 Department of Nutrition, School of Public Health, Sun Yat-sen University, Guangzhou 510080, China; zhouyq79@mail2.sysu.edu.cn (Y.Z.); zengyp27@mail2.sysu.edu.cn (Y.Z.); pangj7@mail2.sysu.edu.cn (J.P.); wangx523@mail2.sysu.edu.cn (X.W.); panzhj5@mail2.sysu.edu.cn (Z.P.); jinyf6@mail2.sysu.edu.cn (Y.J.); cheny2255@mail2.sysu.edu.cn (Y.C.); 2 Guangdong Provincial Key Laboratory of Food, Nutrition and Health, Guangzhou 510080, China; wangrj9@mail2.sysu.edu.cn; 3 Guangdong Engineering Technology Center of Nutrition Transformation, Guangzhou 510080, China; 4 Department of Nutrition, School of Public Health (Shenzhen), Sun Yat-sen University, Shenzhen 518107, China

**Keywords:** resveratrol, hyperuricemia, renal injury, intestinal uric acid metabolism, metagenomics

## Abstract

Resveratrol (RES) has been reported to prevent hyperuricemia (HUA); however, its effect on intestinal uric acid metabolism remains unclear. This study evaluated the impact of RES on intestinal uric acid metabolism in mice with HUA induced by a high-fat diet (HFD). Moreover, we revealed the underlying mechanism through metagenomics, fecal microbiota transplantation (FMT), and 16S ribosomal RNA analysis. We demonstrated that RES reduced the serum uric acid, creatinine, urea nitrogen, and urinary protein levels, and improved the glomerular atrophy, unclear renal tubule structure, fibrosis, and renal inflammation. The results also showed that RES increased intestinal uric acid degradation. RES significantly changed the intestinal flora composition of HFD-fed mice by enriching the beneficial bacteria that degrade uric acid, reducing harmful bacteria that promote inflammation, and improving microbial function via the upregulation of purine metabolism. The FMT results further showed that the intestinal microbiota is essential for the effect of RES on HUA, and that *Lactobacillus* may play a key role in this process. The present study demonstrated that RES alleviates HFD-induced HUA and renal injury by regulating the gut microbiota composition and the metabolism of uric acid.

## 1. Introduction

Hyperuricemia (HUA) has recently become a worldwide public health problem, owing to alterations in lifestyle and diet structure. The global prevalence of HUA has risen significantly, reaching approximately 17.4% in China [1]. HUA is closely associated with a range of metabolic diseases (or metabolic syndromes), including gout, nonalcoholic fatty liver disease (NAFLD), type 2 diabetes, and kidney disease [2]. Therefore, excessive uric acid (UA) levels should be reduced in order to prevent and treat metabolic diseases.

UA is the ultimate oxidized product of the metabolism of purines (adenine and guanine), and HUA is currently thought to be generated via an imbalance between UA production and elimination. The following are the three main pathways that lead to the development of HUA: endogenous overproduction, exogenous (food source) excessive intake, and insufficient excretion [3]. Due to genetic mutations during evolution, humans lack uricase, which oxidizes UA to allantoin, therefore increasing HUA susceptibility. In response to this risk, the microbiota living symbiotically in the human gut may have developed a purine-degrading capacity and excreted UA through the stool. Despite representing one-third of the overall UA excretion, there is a lack of research regarding UA metabolism in the gut. Previous research has indicated that individuals with HUA intestinal microbiota exhibit an imbalance and an increase in the abundance of *Bacteroides*, *Prevotella*, and *Fusobacterium* [4]. Correspondingly, reducing UA can improve the gut microbiota imbalance [5]. In addition to therapeutic measures such as dietary regulation and medication, recent evidence suggests that relatively natural and safe probiotic supplements, herbs, and phytochemicals can successfully prevent UA accumulation, and the underlying mechanism is closely linked to intestinal UA metabolism [6,7]. Consequently, the intestinal flora may be related to HUA pathogenesis and may become a promising therapeutic target for the remission and treatment of HUA.

Resveratrol (RES) is a flavonoid that is found in various foods, such as grapes and wine, berries, including cranberries and red currants, and peanut skins [8]. Numerous preclinical and clinical studies have demonstrated the potential antioxidant, anti-inflammatory [9], cardioprotective, neuroprotective [10], and antiaging effects of RES. Our previous study [11], a double-blind, randomized controlled trial, showed that serum UA and xanthine oxidase (XOD) activity were significantly lowered after eight weeks of 600 mg/day RES intervention in individuals with dyslipidemia than following placebo treatment. Additionally, a strong relationship was found between alterations in UA and XOD activity. However, whether RES reduces UA levels via gut microbes remains to be explored.

A high-fat diet (HFD) is linked to various digestive, cardiovascular, and urinary diseases, including HUA [12], which accelerates the progression of diseases by causing inflammation and metabolic imbalances [13]. In this research, an HFD-induced model of HUA was utilized in order to mimic human metabolic disorders more accurately, and the preventive impact of RES on HUA was assessed using biochemical markers and histopathological analysis. Metagenomic analysis was subsequently used to further explore the critical molecules associated with HUA. Fecal microbiota transplantation (FMT) and 16S ribosomal RNA (rRNA) sequencing were ultimately employed to investigate whether the gut microbiota plays a crucial role in the UA-lowering benefits of RES.

## 2. Materials and Methods

### 2.1. Design of the Intervention Experiments

Thirty-six-week-old C57BL/6J male mice were purchased from the Guangdong Medical Laboratory Animal Center (Foshan, China), and then split into three groups: one on a low-fat diet (LFD group), another on an HFD (HFD group), and the last one receiving RES intervention (HFD + RES group), all for a duration of eight weeks. Only male mice were used because female mice fed a high-fat diet did not exhibit overt HUA, despite obesity and hepatic steatosis, which may possibly be related to fluctuations in secreted hormones [14]. The LFD group consumed a standard diet (D12450J, Research Diet, New Brunswick, NJ, USA), the HFD group consumed an HFD (D12492, Research Diet, New Brunswick, NJ, USA), and the HFD + RES group consumed an HFD supplemented with RES (0.1% in diet, Mega Resveratrol, Danbury, CT, USA). This dose of RES refers to 600 mg/day in our previous randomized controlled trial [11], which translates to approximately 100 mg/kg/day in mice based on the body surface area of humans and mice [15]. The mice were given fresh feed every 3 days, and their food intake was recorded. The amount of water, levels of activity, and the health of the mice were observed daily, and their weight was measured once a week. Fresh feces were collected each day for the last two weeks in preparation for subsequent FMT. Urine was collected over a 24 h period in a metabolic cage on the day prior to the end of the experiment. Finally, all mice were euthanized via carbon dioxide anesthesia and cervical dislocation, and blood, cecal contents, liver, and kidneys were collected.

### 2.2. Fecal Microbiota Transplantation

FMT experiments aimed to identify the gut microbiota as a critical factor. Newly excreted feces were collected from the mice (donor mice) and cryopreserved in the HFD and HFD + RES groups during the intervention study. After thawing, the feces were homogenized in phosphate-buffered saline (PBS) containing 0.05% cysteine HCl in order to prepare a 200 mg/mL fecal slurry, which was filtered through a 100 μm strainer and then packaged and frozen at −80 °C. The slurry was incubated at 37 °C to resuscitate the microflora until use for transplantation. Mice that received fecal microbiota from HFD-fed mice were labeled the HFD-FMT group, while mice that received fecal microbiota from the HFD + RES group were labeled the HFR-FMT group. Both groups were maintained on an HFD during the study period. Prior to the initial transplantation, they were provided with water containing PEG3350 (17 mmol/L), and they fasted overnight to remove gut bacteria [16]. Transplantation (200 μL) was performed every 2 days for 4 weeks, a total of 14 times.

### 2.3. Metagenomic Sequencing

Total DNA was extracted from fecal samples using the hexadecyl trimethyl ammonium bromide (CTAB) method (LC-Biotechnology Co., Ltd., Hangzhou, China), and the quality of the extracted DNA was determined via agarose gel electrophoresis. The DNA was quantified via an ultraviolet spectrophotometer. A paired-end library was constructed through the use of a specific kit. Following library qualification, high-throughput sequencing was carried out using a NovaSeq 6000 with the PE150 sequencing mode. Library construction involves disrupting genomic DNA using ultrasonication, repairing DNA fragments, adding an ‘A’ base to the 3′ end of the fragment, splicing sequencing adapters, selecting fragments, and amplifying using a polymerase chain reaction (PCR). After data analysis, the sequencing data were processed in order to remove joint and low-quality sequence data. After data preprocessing, a single sample was subjected to de novo assembling. The assembled contigs were predicted using coding sequences, and short contig sequences were filtered out. A non-redundant unigene set was obtained via sequence clustering. The unigenes were matched against the NR_meta database in order to gather taxonomic details about the species at various taxonomic ranks. Alpha and beta diversity assessments were performed using the statistical findings that were individually annotated for each species. Annotation information was obtained by comparing the unigenes using Gene Ontology (GO) and Kyoto Encyclopedia of Genes and Genomes (KEGG) functional databases. The abundance and differences in the unigenes were analyzed at the species classification, functional annotation, and unigene levels. Additionally, the enrichment analysis of the unigenes was performed using the GO and KEGG databases.

### 2.4. 16S rRNA Gene Sequencing

Total DNA was extracted from the fecal samples using the CTAB method (LC-Biotechnology Co., Ltd., Hangzhou, China), after which PCR amplification, purification, and quantification were performed. The quality of the PCR products was assessed with an Agilent 2100 Bioanalyzer (Agilent, Palo Alto, CA, USA) and an Illumina library quantification kit (Kapa Biosciences, Woburn, MA, USA). A NovaSeq 6000 SP Reagent Kit (500 cycles) was used for double-ended sequencing. After on-machine sequencing, the original off-machine data and raw data were obtained. Overlap was employed for splicing the double-end data, followed by quality control and chimera filtering to acquire high-quality and clean data. We obtained a feature table and sequence following dereplication using adenosine deaminase 2 deficiency. Alpha and beta diversity assessments were performed using the abundance table of the acquired amplicon sequence variants (ASVs). Alpha diversity analysis mainly evaluates the domestic diversity through Simpson and Chao1 indices. Four distances (weighted UniFrac, unweighted UniFrac, Jaccard, and Bray–Curtis) were used to evaluate the beta diversity among the habitats (samples/groups). The ASV (feature) sequence file was generated using the SILVA (Release 138) database in conjunction with the NT-16s annotation database for species. The quantity of every type of organism in each sample was examined using the ASV (feature) abundance chart. The analysis of variations between the comparison groups was based on the statistical data regarding species abundance.

### 2.5. Biochemical Parameter Detection

The manufacturer’s instructions for measuring serum UA, creatinine (CRE), blood urea nitrogen (BUN), and urine UA and CRE were followed using a Cobas c311 automatic analysis analyzer (c311, Roche Diagnostics, Basel, Switzerland). Urinary protein levels were detected with commercial kits (Cat. NO. E-BC-K252-M, Elabscience, Wuhan, China). The fecal and cecal contents were homogenized in cold normal saline and centrifuged at 3000 revolutions per minute (rpm) for 10 min, after which the supernatant was collected. The levels of UA (Cat. NO. E-BC-K016-M) and urea (Cat. NO. E-BC-K183-M) in the feces and cecal contents were measured using commercial kits (Elabscience, Wuhan, China).

### 2.6. XOD Activity Detection

After the mouse liver tissue was homogenized in cold normal saline and centrifuged at 3000 rpm for 10 min, the supernatant was collected. The levels of hepatic and serum XOD activity were measured using commercial kits (Cat. NO. E-BC-K805-M, Elabscience, Wuhan, China).

### 2.7. Renal Histological Analysis

Renal pathology included hematoxylin-eosin (HE), Masson’s trichrome, and periodic acid-Schiff (PAS) staining. The mouse kidneys were fixed in 4% paraformaldehyde for 48 h. The fixed kidney tissue was embedded in paraffin following standard dehydration, transparency, and wax immersion procedures. Five-micron-thick tissue sections were prepared following the standard procedure for staining. The images were viewed under a light microscope (Nikon Eclipse E100, Nikon, Tokyo, Japan) at 400× magnification. ImageJ software (version 1.43, National Institutes of Health, Bethesda, MD, USA) was used to measure the collagen content and PAS-positive area in the kidney tissue samples.

### 2.8. Enzyme-Linked Immunosorbent Assay (ELISA)

Kidney tissue was rinsed with a precooled PBS to remove any residual blood. The kidney tissue slices, and PBS were combined in a homogenizer, ground thoroughly on ice, and then centrifuged at 2500 rpm for 10 min in order to obtain the supernatant for detection. Renal tumor necrosis factor-α (TNF-α, Cat. NO. E-EL-M3063, Elabscience, Wuhan, China), interleukin-6 (IL-6, Cat. NO. E-EL-M0044c, Elabscience, Wuhan, China), interleukin-1β (IL-1β, Cat. NO. E-EL-M0037c, Elabscience, Wuhan, China), fecal lipopolysaccharides (LPS, Cat. NO. JL20691, J&L Biological, Shanghai, China), and a serum lipopolysaccharide-binding protein (LBP, Cat. NO. E-EL-M3090, Elabscience, Wuhan, China) were measured using commercial ELISA kits.

### 2.9. Determination of the UA Degradation Capacity of Cecum Contents

The UA degradation capacity was determined as previously described with minor modifications [17]. Approximately 0.1 g of cecal content was measured, combined with 400 μL of sterile PBS, agitated for 5 min, and subsequently centrifuged at 3000 rpm for 10 min. After the supernatant was collected, it was combined with 10 μL of a 6 mM UA standard solution and incubated in a mixing tube at 37 °C for 6 h. Following the incubation, the mixture was centrifuged at 15,000 rpm for 15 min, resulting in the collection of the supernatant. The UA concentration before and after the incubation of the supernatant was then measured using commercial kits (Cat. NO. C012-1-1, JianCheng, Nanjing, China). The UA degradation capacity was expressed as the difference of UA concentration before and after incubation. The larger the difference is, the stronger the degradation capacity, and vice versa.

### 2.10. Statistical and Bioinformatics Analysis

Statistical analyses were performed with GraphPad Prism (version 9.0, GraphPad Software, Boston, MA, USA). When the dataset showed a normal distribution and homoscedasticity, Student’s *t* test and one-way ANOVA were applied; otherwise, the Kruskal–Wallis test was used. The data are expressed as the mean ± standard deviation (SD). A *p* value < 0.05 was considered to indicate a significant difference.

## 3. Results

### 3.1. Effect of RES on HFD-Induced HUA

**Figure 1:**
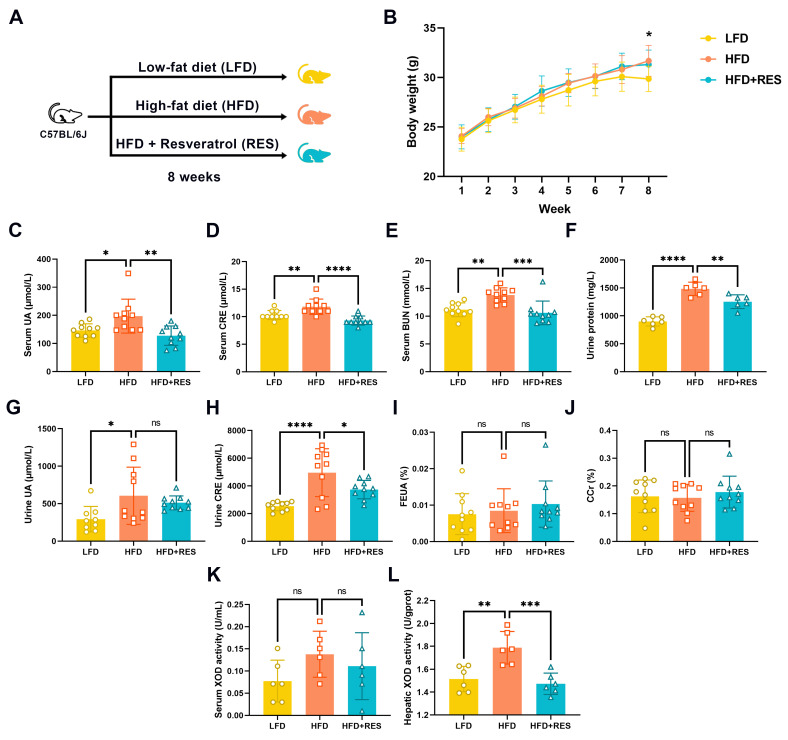
Effect of resveratrol on HFD-induced hyperuricemia. (**A**) Diagram illustrating the experimental procedures. (**B**) Changes in the body weights of the mice after eight weeks of treatment (*n* = 10). * *p* < 0.05 among the three groups of body weight. Serum uric acid (**C**), creatinine (**D**), urea nitrogen (**E**), and urine protein (**F**) levels of the mice; *n* = 6–10. Urine uric acid (**G**), creatinine (**H**), fraction excretion of uric acid (**I**), and creatinine clearance rate (**J**) of the mice (*n* = 9–10). Serum (**K**) and hepatic (**L**) xanthine oxidase activity in mice (*n* = 6). The data are presented as the mean ± SD and were analyzed using either ANOVA or the Kruskal–Wallis test. * *p* < 0.05, ** *p* < 0.01, *** *p* < 0.001, and **** *p* < 0.0001.

To clarify the impact of RES on HUA, C57BL/6J mice were fed an HFD for eight weeks to induce HUA development, as illustrated in Figure 1A. The serum and urine UA levels of the HFD-fed mice were markedly greater than those of the LFD-fed mice (both *p* < 0.05; Figure 1C,G). However, RES supplementation resulted in lower serum UA levels (*p* < 0.01; Figure 1C). Furthermore, RES improved other parameters related to renal function, as demonstrated by the notable reductions in serum CRE (*p* < 0.0001; Figure 1D), BUN (*p* < 0.001; Figure 1E), and urinary protein levels (*p* < 0.01; Figure 1F), and the significant decrease in urine CRE (*p* < 0.05; Figure 1H). However, we failed to observe significant changes in the concentration of urine UA, the fractional excretion of uric acid, or the creatinine clearance rate. RES also inhibited hepatic XOD activity (*p* < 0.001; Figure 1L), whereas there was no marked change in serum XOD activity.

### 3.2. Effect of RES on Renal Injury and Inflammation

**Figure 2:**
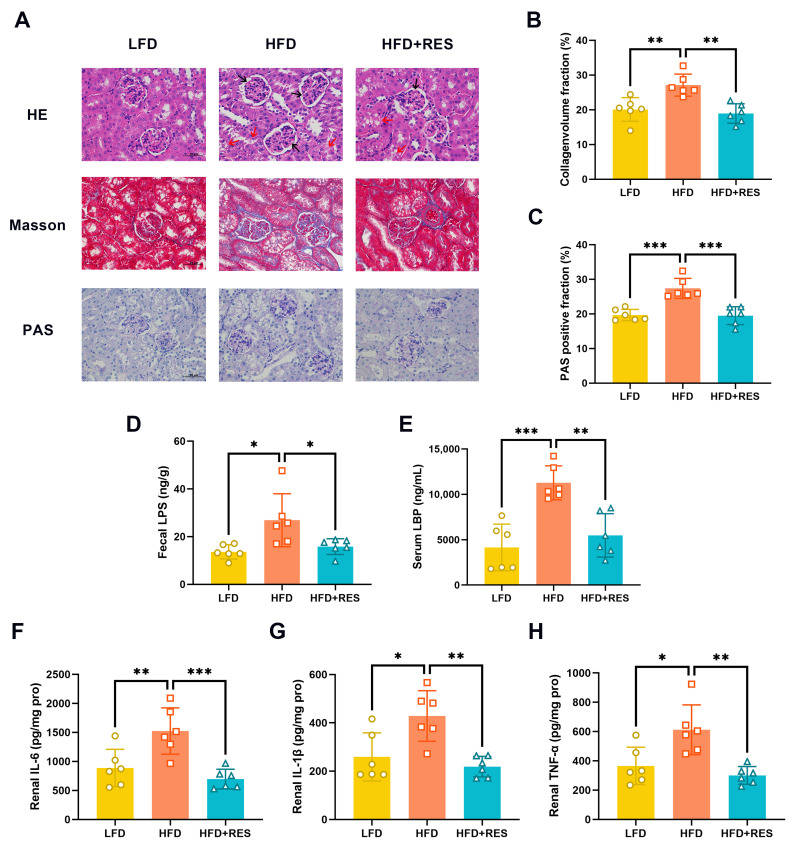
Effect of resveratrol on renal injury and inflammation. (**A**) Representative HE, Masson’s trichrome, and PAS staining of kidney tissue sections (scale bars = 50 μm). Magnification ×400. The black arrows indicate glomerular atrophy, and the red arrows indicate unclear renal tubule structure. Quantitative analysis of the collagen volume fraction (**B**) and PAS-positive fraction (**C**) of the kidney tissue sections (*n* = 6). Levels of fecal LPS (**D**), serum LBP (**E**), renal IL-6 (**F**), IL-1β (**G**), and TNF-α (**H**) in mice (*n* = 6). The data are presented as the mean ± SD and were analyzed using either ANOVA or the Kruskal–Wallis test. * *p* < 0.05, ** *p* < 0.01, and *** *p* < 0.001.

HE staining of the kidneys revealed normal renal tissue in the LFD group. In contrast, the HFD group exhibited glomerular atrophy and an unclear renal tubule structure with obvious vacuolar degeneration (Figure 2A). Masson’s trichrome and PAS staining demonstrated collagen fiber deposition and glycogen accumulation, respectively. The results indicated that the HFD group had significantly greater levels of collagen fibers and glycogen than the LFD group (Figure 2B,C). However, RES intervention effectively ameliorated HFD-induced glomerular atrophy, an unclear renal tubule structure (Figure 2A), renal fibrosis (*p* < 0.05; Figure 2B), and glycogen deposition (*p* < 0.01; Figure 2C).

Furthermore, the ELISA results suggested that RES significantly reduced the concentrations of fecal LPS (*p* < 0.05; Figure 2D), serum LBP (*p* < 0.01; Figure 2E), and renal proinflammatory factors, including IL-6 (*p* < 0.001; Figure 2F), IL-1β (*p* < 0.01; Figure 2G), and TNF-α (*p* < 0.01; Figure 2H), in HFD-fed mice.

### 3.3. Effect of RES on Intestinal UA Metabolism

**Figure 3:**
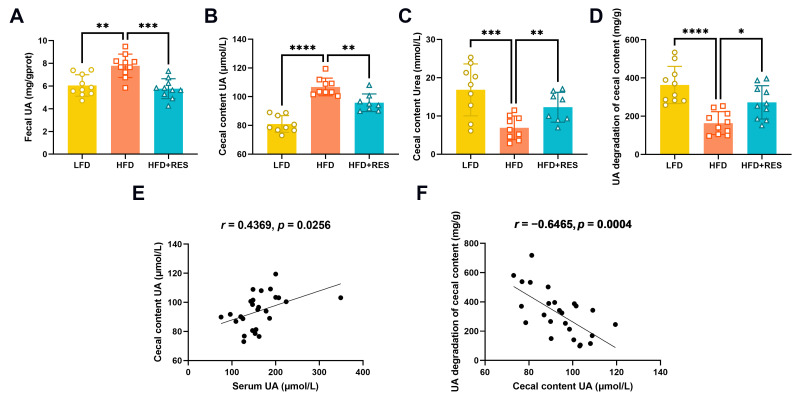
Effect of resveratrol on intestinal uric acid metabolism. Fecal uric acid (**A**), cecal content uric acid (**B**), cecal content urea (**C**), and uric acid degradation in the cecal contents of mice (**D**); *n* = 8–10. (**E**) Correlation between the levels of serum uric acid and cecal content uric acid in the mice (*n* = 26). (**F**) Correlation between the levels of uric acid and its degradation in the cecal contents of the mice (*n* = 26). The data are presented as the mean ± SD and were analyzed using either ANOVA or the Kruskal–Wallis test. * *p* < 0.05, ** *p* < 0.01, *** *p* < 0.001, and **** *p* < 0.0001.

The UA levels in the feces (*p* < 0.01; Figure 3A) and cecal contents (*p* < 0.0001; Figure 3B) were significantly increased when fed a HFD, while the levels of urea, a metabolite of UA degradation, were decreased (*p* < 0.001; Figure 3C). However, these trends were reversed by RES (Figure 3A–C). Additionally, the serum UA levels were positively correlated with the UA levels in the cecal contents (*p* < 0.05; Figure 3E). To investigate the effect on UA degradation, we measured the UA levels in the cecal contents before and after incubation. The results indicated that HFD-fed mice supplemented with RES exhibited increased an UA degradation capacity (*p* < 0.05; Figure 3D). Interestingly, a marked negative correlation was found between the UA concentration and UA degradation ability in the cecum contents (*p* < 0.001; Figure 3F). The results indicate that RES could lower serum UA levels via improving the capacity for UA degradation in the intestines.

### 3.4. Effects of RES on the Structural Dysregulation of the Intestinal Flora

**Figure 4:**
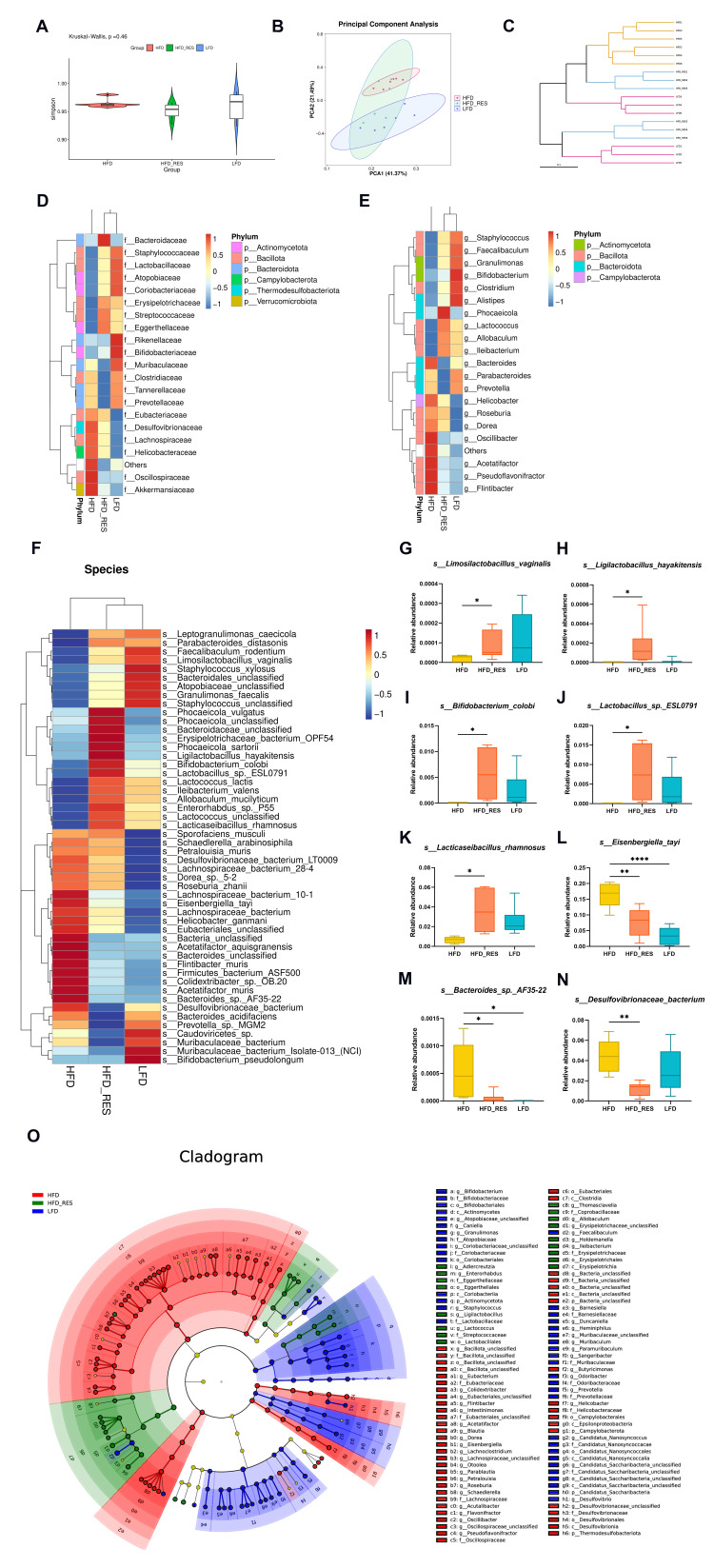
Effects of resveratrol on the structural dysregulation of the intestinal flora. (**A**) α-Diversity of gut bacteria. (**B**) PCA of microbial communities. (**C**) Hierarchical cluster analysis. (**D**) Heatmap presenting the top 20 most abundant bacteria at the family level. (**E**) Heatmap showing the top 20 most abundant bacteria at the genus level. (**F**) Heatmap displaying the relative abundance of bacteria at the species level and boxplots (**G**–**N**) demonstrating the abundance of selected species with significant differences between groups. * *p* < 0.05, ** *p* < 0.01, and **** *p* < 0.0001. (**O**) Cladogram illustrating bacteria with significant differences among groups according to linear discriminant analysis (LDA) effect size (LEfSe) analysis, with LDA scores > 3.0 and adjusted *p* < 0.05. The gradient from blue to red reflects the change in abundance from low to high. *n* = 6 per group.

We conducted metagenomic sequencing to assess the effect of RES on the gut microbiota by examining alterations in its composition and function. Initially, the diversity within the intestinal microbiota of the LFD group was greater than that of the other two groups based on the Simpson index, but these differences did not reach statistical significance (Figure 4A). Principal component analysis (PCA) demonstrated noticeable separation between the LFD and HFD groups, indicating high dissimilarity among the intestinal flora. Notably, the intestinal flora composition of the HFD + RES group overlapped with those of the other two groups (Figure 4B). Consistently, the cluster tree showed that the HFD and LFD groups were far apart, while the HFD + RES and LFD groups were closer together (Figure 4C), suggesting that RES treatment-induced alterations in the intestinal flora composition of HFD-fed mice tended to normalize.

The classification and composition of the intestinal microbiota were further analyzed. Based on the abundance of the top 20 differentiated families when compared with those in the HFD group, the relative abundances of *Lactobacillaceae*, *Erysipelotrichaceae*, *Streptococcaceae*, and *Eggerthellaceae* in the RES group tended to increase. In contrast, the relative abundances of *Prevotellaceae*, *Desulfovibrionaceae*, *Lachnospiraceae*, and *Helicobacteraceae* decreased (Figure 4D). At the genus level, there was a rising trend in the abundance of *Faecalibaculum*, *Bifidobacterium*, *Clostridium*, and *Lactococcus* in the HFD + RES group. In contrast, the abundances of *Bacteroides*, *Helicobacter*, and *Prevotella* showed the opposite trend (Figure 4E).

The advantage of metagenomic sequencing is that it can identify microbiota at the species level. As shown in the heatmap of cluster analysis at the species level (Figure 4F), the abundances of many probiotics, such as *Lactobacillus_*sp.*_ESL0791*, *Lacticaseibacillus_rhamnosus*, *Bifidobacterium_colobi*, *Ligilactobacillus_hayakitensis*, and *Limosilactobacillus_vaginalis*, were notably greater in the HFD + RES group than in the HFD group (Figure 4G–K). In contrast, the abundances of opportunistic pathogens, including *Eisenbergiella_tayi*, *Desulfovibrionaceae_bacterium*, and *Bacteroides_*sp.*_AF35-22*, decreased significantly (Figure 4L–N).

Linear discriminant analysis effect size (LEfSe) analysis was employed to identify the dominant microorganisms within each group. The results demonstrated a marked enrichment of probiotics, such as *o_Lactobacillales*, *g_Ligilactobacillus*, and *o_Eggerthellales*, in the HFD + RES group (Figure 4O).

### 3.5. Effect of RES on the Function of the Gut Microbiota

**Figure 5:**
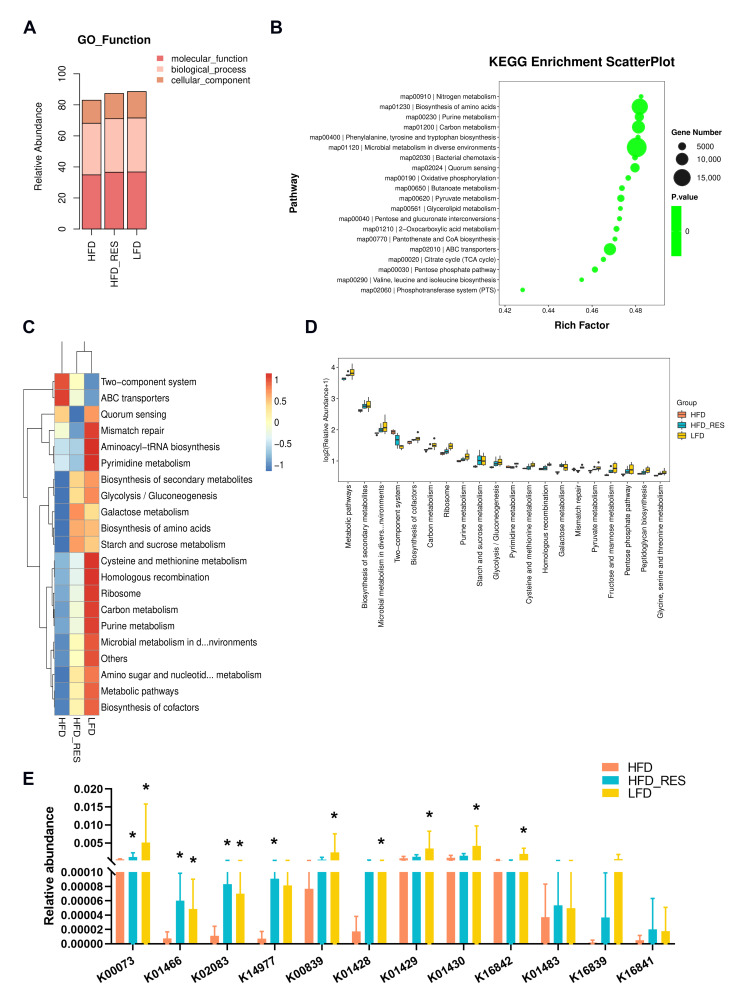
Effects of resveratrol on functional changes in the gut microbiota. (**A**) Stacked bars demonstrate the abundance of each category in the GO database. (**B**) The KEGG scatter plot displays the top 20 pathways with significant group differences. (**C**) Heatmap displaying the top 20 pathways in the KEGG database. (**D**) The box plot depicts the top 20 pathways with significant differences among groups in the KEGG database. (**E**) Relative abundance of KOs involved in uric acid metabolism based on the KEGG database. The Wilcoxon rank-sum test revealed significant differences compared to those in the HFD group, and the results are indicated by * *p* < 0.05.

To investigate the regulatory effect of RES on the function of the gut microbiota related to UA metabolism, we used the GO and KEGG databases for the functional annotation of the metagenomic data. The GO database annotation findings showed that, when compared with the HFD group, the HFD + RES group had a greater gene abundance in terms of molecular function, biological process, and cellular component functions, which tended to be more similar to those in the LFD group (Figure 5A). The top 20 pathways according to the enrichment data are listed in the KEGG enrichment scatter plot, which shows significant enrichment of the purine metabolic pathways (Figure 5B). In addition, an analysis of KEGG pathways revealed a decrease in the expression of genes related to purine metabolism in the HFD group when compared to the LFD group, and RES treatment reversed this disruption (Figure 5C,D), indicating that the altered pathways in the RES group were associated with purine metabolism.

The univariate analysis of the related coding genes (KOs) involved in the UA degradation pathway was performed. Compared to those in the LFD group, the abundances of twelve KOs (K00073: allD, ureidoglycolate dehydrogenase; K01466: allB, allantoinase; K02083: allC, allantoate deiminase; K14977: ylbA, (S)-ureidoglycine aminohydrolase; K00839: pucG, (S)-ureidoglycine; K01428: ureC, urease subunit alpha; K01429: ureB, urease subunit beta; K01430: ureA, urease subunit gamma; K16842: hpxB, allantoinase; K01483: allA, ureidoglycolate lyase; K16839: hpxO, FAD-dependent urate hydroxylase; K16841: hpxA, allantoin racemase) involved in UA degradation in the HFD group were downregulated, of which eight showed significant differences, while four were significantly increased following RES intervention (Figure 5E). These findings indicated that RES improved functional modifications in the gut microbiota of HFD-fed mice, which included the pathways involved in UA degradation.

### 3.6. The Intestinal Flora Is the Key to RES-Mediated HUA Regulation

**Figure 6:**
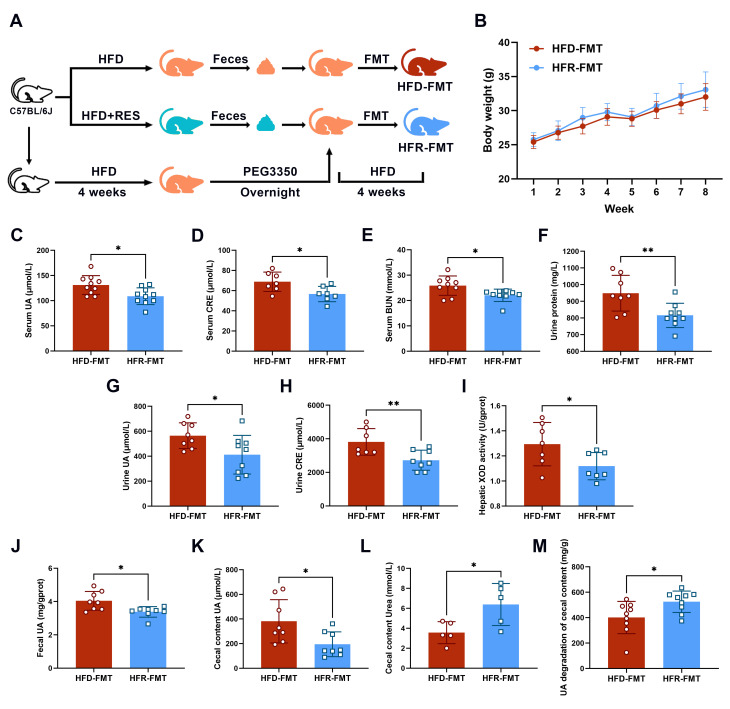
Effects of fecal microbiota transplantation on HFD-induced hyperuricemia. (**A**) Diagram illustrating the experimental procedures. (**B**) Changes in the body weights of the mice after eight weeks of treatment. Serum uric acid (**C**), creatinine (**D**), urea nitrogen (**E**), and urine protein (**F**) levels in the mice. Urine uric acid (**G**), creatinine (**H**), and hepatic xanthine oxidase activity (**I**) in the mice (*n* = 7–10). Fecal uric acid (**J**), cecal content uric acid (**K**), cecal content urea (**L**), and uric acid degradation in the cecal contents (**M**) of the mice (*n* = 5–9). The data are presented as the mean ± SD and were analyzed using either ANOVA or the Kruskal–Wallis test. * *p* < 0.05 and ** *p* < 0.01.

To explore the role of the gut microbiota in the UA-lowering benefits of RES, we conducted FMT following the intervention experiment (Figure 6A). The findings indicated the following: a more favorable trend in the HFR-FMT group toward reducing serum UA (*p* < 0.05; Figure 6C), CRE (*p* < 0.05; Figure 6D), and BUN levels (*p* < 0.05; Figure 6E); decreasing urinary protein (*p* < 0.01; Figure 6F), urine UA (*p* < 0.05; Figure 6G), and CRE (*p* < 0.01; Figure 6H); inhibited hepatic XOD activity (*p* < 0.05; Figure 6I); and the promoting of intestinal UA degradation (*p* < 0.05; Figure 6J–M), when compared to those in the HFD-FMT group. 

**Figure 7:**
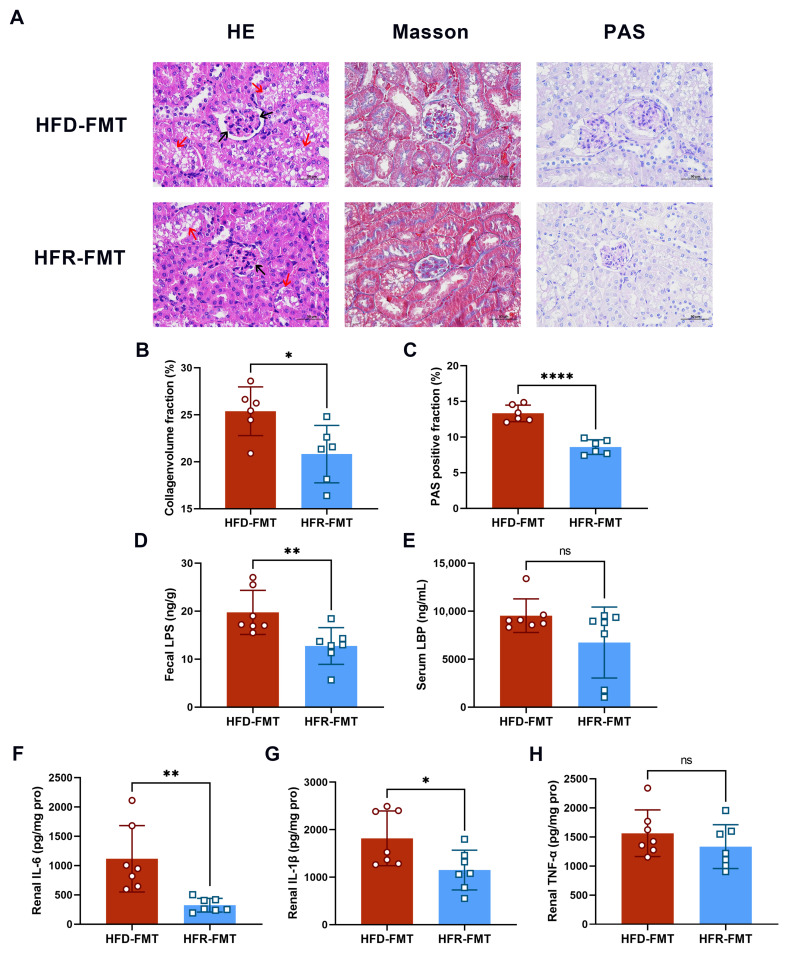
Effects of fecal microbiota transplantation on renal injury and inflammation. (**A**) Representative HE, Masson’s trichrome, and PAS staining of kidney tissue sections (scale bars = 50 μm). Magnification ×400. The black arrows indicate glomerular atrophy, and the red arrows indicate unclear renal tubule structure. Quantitative analysis of the collagen volume fraction (**B**) and PAS-positive fraction (**C**) of the kidney tissue sections (*n* = 6). Levels of fecal LPS (**D**), serum LBP (**E**), renal IL-6 (**F**), IL-1β (**G**), and TNF-α (**H**) in mice (*n* = 7). The data are presented as the mean ± SD and were analyzed using either ANOVA or the Kruskal–Wallis test. * *p* < 0.05, ** *p* < 0.01, and **** *p* < 0.0001.

According to renal histological analysis, the HFR-FMT group exhibited less glomerular atrophy and a clearer renal tubule structure (Figure 7A), and renal fibrosis (*p* < 0.05; Figure 7B) and glycogen deposition (*p* < 0.0001; Figure 7C) were also less severe in this group. In addition, the levels of fecal LPS (*p* < 0.01; Figure 7D), serum LBP (Figure 7E), and renal proinflammatory factors, including IL-6 (*p* < 0.01; Figure 7F), IL-1β (*p* < 0.05; Figure 7G), and TNF-α (Figure 7H), were decreased in the HFR-FMT group, although there were no significant differences between the reduction in serum LBP or renal TNF-α. These results suggested that the intestinal microbiota is vital for mediating the improvement of the effects of RES on HUA.

**Figure 8:**
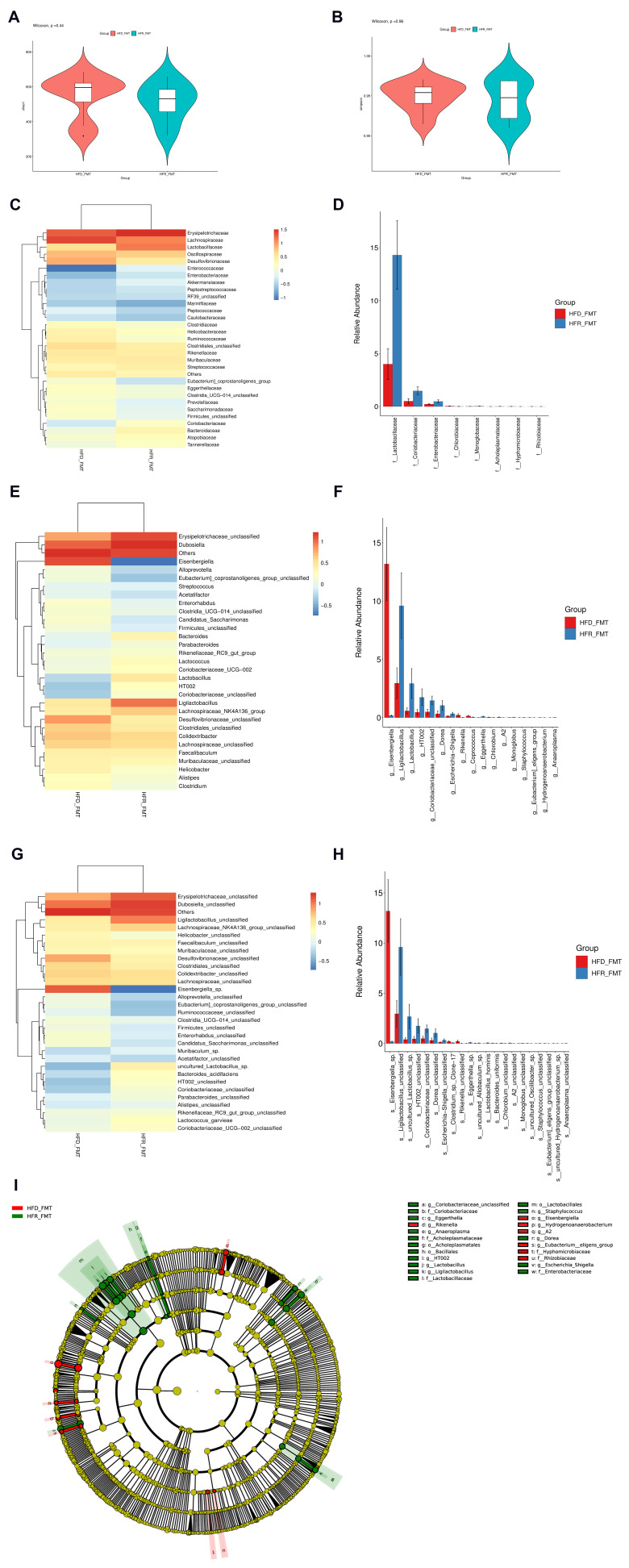
Effects of fecal microbiota transplantation on intestinal flora. (**A**) Chao1 index. (**B**) Simpson index. (**C**) Heatmap illustrating the top 30 most abundant bacteria at the family level. (**D**) Box plot describing the bacteria that differed significantly at the family level between groups. (**E**) Heatmap presenting the top 30 most abundant bacteria at the genus level. (**F**) Box plot showing the bacteria that differed significantly at the genus level between groups. (**G**) Heatmap demonstrating the top 30 most abundant bacteria at the species level. (**H**) Box plots displaying the bacteria differed significantly between the groups at the species level. (**I**) Cladogram manifesting bacteria with significant differences among groups according to LEfSe analysis with LDA scores > 3.0 and adjusted *p* < 0.05. The gradient from blue to red reflects the change in abundance from low to high (*n* = 8) per group.

We further analyzed the gut microbiome of the mice subjected to FMT via 16S rRNA gene sequencing. Initially, there were no notable differences in the diversity of the gut bacteria between the two groups, as shown by the Chao1 and Simpson indices (Figure 8A,B). At the family level, the relative abundance of *Lactobacillaceae*, *Coriobacteriaceae*, and *Enterobacteriaceae* was significantly greater in the HFR-FMT group than in the HFD-FMT group (Figure 8C,D). At the genus level, there were significantly greater relative abundances of *Ligilactobacillus*, *Dorea*, *Escherichia-Shigella*, and *Eggerthella,* and lower relative abundances of *Eisenbergiella* and *Rikenella* in the HFR-FMT group (Figure 8E,F). At the species level, *Ligilactobacillus_unclassified*, *Escherichia-Shigella_unclassified*, *Lactobacillus_hominis*, and *Lactobacillus_intestinalis* were more abundant in the HFR-FMT group than in the HFD-FMT group, whereas *Eisenbergiella_*sp. and *Rikenella_unclassified* were substantially lower (Figure 8G,H).

LEfSe analysis revealed a marked predominance of *g_Ligilactobacillus* and *o_Lactobacillales* in the HFR-FMT group, while the abundance of *g_Eisenbergiella* were greater in the HFD-FMT group (Figure 8I).

The LEfSe analysis of metagenomic sequencing and 16S rRNA revealed that *o_Lactobacillales* and *g_Ligilactobacillus* were common biomarkers in the RES intervention and FMT groups, suggesting that *Lactobacillus* is vital for the effect of RES.

## 4. Discussion

Epidemiological studies have shown that a disturbed gut microbiota is crucial for HUA pathological development [6,18,19]. Although clinical [11,20] and preclinical studies [21,22] have confirmed the UA-lowering effects of RES, whether these effects can be attributed to the gut microbiota remains to be determined. We used metagenomic and 16S rRNA analyses to assess how RES reduces UA levels and the underlying mechanism in HFD-fed mice. Our findings indicate that RES has the potential to alleviate HFD-induced HUA and renal injury, as indicated by reduced serum UA, CRE, BUN, and urinary protein levels and ameliorated nephritis and fibrosis. The underlying mechanism by which RES improves HUA may involve regulating the intestinal microbiota composition and function, and promoting UA degradation via the intestinal microbiota.

A long-term HUA could increase the concentration of UA in the kidney and form urate crystals, which recruit a large number of inflammatory cytokines, leading to renal injury. Therefore, controlling the level of serum UA within the normal range will help alleviate renal injury. Studies have shown that HFD feeding can lead to HUA and chronic kidney injury in mice, the specific pathological manifestations of which are glomerular atrophy, the proliferation of mesangial cells and mesangial matrix, unclear renal tubule structure, fibrosis, and chronic inflammation [23,24]. In this study, HFD-fed mice treated with RES had less glomerular atrophy and clearer renal tubule structures, as observed by HE staining, while the areas of Masson and PAS-positive staining were reduced, indicating decreased renal fibrosis and mesangial matrix proliferation, respectively. In addition, combined with the lower levels of kidney inflammatory factors, RES ameliorated the renal injury induced by HFD, which is consistent with previous studies [25,26]. It is worth mentioning that the intervention dose of RES used in this experiment was converted from the effective dose of 600 mg/day in a previous trial to the corresponding 100 mg/kg/day in mice, which has also been applied in interventions for a variety of disease models, such as insulin resistance [25], obesity [27], and cognitive impairment [28], all with good tolerance and safety.

RES has been shown to improve HUA in both animal studies and clinical trials via the inhibition of XOD activity and the regulation of organic ion transporters in the kidney and ileum [29,30]. Liang et al. demonstrated that rhizoma smilacis glabrae extracts rich in RES decreased serum UA levels and inhibited liver XOD activity in HUA mice [31]. Shi et al. reported that 40 mg/kg/day of RES significantly reduced the levels of serum UA and renal UA transporters, including OAT1, OAT3, and URAT1 [32]. Similarly, Zhang et al. established a mouse model of insulin resistance combined with HUA induced by HFD, and reported that RES at a dose of 100 mg/kg/day improved inflammation and reduced UA transporter expression in the kidney [25]. Our previous clinical trial showed that RES effectively reduced the levels of serum UA and XOD activity in a dose-dependent manner in participants with dyslipidemia [11]. Moreover, it has been reported that RES dimerization can reduce UA levels in healthy subjects, and the mechanism has been confirmed through in vitro experiments that might be linked to the suppression of angiotensin activity in order to prevent metabolic waste reabsorption in the renal tubular epithelium [20,33]. These results reveal that RES may reduce UA through multiple targets; however, no studies have investigated the mechanism of RES in intestinal UA metabolism.

The gut microbiota is essential for maintaining host physiological function [34,35,36], and its dysregulation is closely related to metabolic diseases in humans [37,38], including UA metabolism and the occurrence and development of HUA [39,40]. It is known that the intestinal flora promotes the metabolism of purines and UA [41]. According to a study by He et al. [17], inulin-type prebiotics were found to lower serum UA levels in patients with end-stage renal disease by increasing the degradation of UA in the intestinal microbiota. Studies have revealed that the ribonucleoside hydrolase in *Lactobacillus* can hydrolyze nucleosides into nucleobases in order to reduce the serum UA level in mice via the degrading of purine nucleosides and reducing purine absorption in the intestine [42,43].

Moreover, *Lactobacillus* and *Pseudomonas* are reported to produce UA-metabolizing enzymes, such as uricase, allantoinase, and allantoicase, which convert UA into 5-hydroxyisourate, allantoin, allantoate, and finally urea [44]. The present study is the first to show that RES significantly reduced UA in the serum, feces, and cecal contents of HFD-fed mice, as well as increasing cecal urea. Moreover, the greater capacity for UA degradation in the cecum contents and its significant negative correlation with the serum UA concentration suggested that RES could lower serum UA levels by enhancing intestinal UA degradation and reducing the UA concentration in feces. Accordingly, RES caused significant changes in the gut microbiota structure of HFD-fed mice, thus affecting various taxonomic levels, including family, genus, and species. RES greatly increased the abundance of *Lactobacillaceae*, *Lactobacillales*, and *Lactobacillus_*sp.*_ESL0791*, which are reportedly involved in the catabolism of UA.

Furthermore, chronic inflammation is a common characteristic of HUA [45], and the intestinal flora may be involved in anti-inflammatory and proinflammatory mechanisms in HUA [46]. Many beneficial bacteria, such as *Bifidobacterium* [47], *Lactobacillus* [48], and *Erysipelotrichaceae* [49], are known to reduce inflammation. In a high fructose-induced HUA model, *Lactobacillus brevis DM9218* reduced inflammatory cytokine-stimulated XOD activity by degrading inosine, an intermediate metabolite in the gut, thereby reducing serum UA levels [50]. Similarly, Zhao et al. [51] reported that *Lacticaseibacillus rhamnosus Fmb14* mitigated inflammatory responses, reduced liver XOD and kidney URAT1 levels, and alleviated renal fibrosis in chronic purine-induced HUA mice. Consistent with these studies, we found that RES reduced the increased concentrations of key proinflammatory factors in the kidneys, such as IL-6, IL-1β and TNF-α; inhibited hepatic XOD activity; and increased the abundances of *Bifidobacterium colobi*, *Ligilactobacillus hayakitensis*, *Limosilactobacillus vaginalis*, *Erysipelotrichaceae,* and *Lacticaseibacillus rhamnosus*. In addition, short-chain fatty acid (SCFA), a metabolite of the intestinal flora, has been shown to ameliorate renal inflammation and promote uric acid excretion by providing energy to enterocytes, enhancing the epithelial barrier function, and inhibiting the release of proinflammatory cytokines [51,52,53]. In the present study, we found that RES treatment increased the abundance of SCFA-producing genera, such as *Clostridium* [54], *Bifidobacterium*, and *Faecalibaculum* [55,56], accompanied by a decrease in renal proinflammatory cytokines.

On the other hand, an increase in pathogenic bacteria such as endotoxin-producing *Desulfovibrionaceae* [57,58], *Bacteroides* [59], and *Helicobacteraceae* [49] is probably responsible for the increased levels of circulating LPS and proinflammatory cytokines, leading to metabolic inflammation. Our research revealed a decrease in the levels of fecal LPS and serum LBP after RES intervention and a reduction in the abundances of *Desulfovibrionaceae bacterium*, *Bacteroides sp. AF35-22*, and *Helicobacteraceae*, suggesting that a reduction in the abundance of harmful bacteria may contribute to lowering the inflammation induced by HFD.

A recently published large metagenomic study reported that the intestinal flora of gout patients displayed a greater abundance of genes associated with carbohydrate and energy metabolism, while healthy controls had relatively more UA degradation genes [4]. Consistent with the alteration in the UA degradation capacity of cecum contents, our metagenomic analysis revealed that RES significantly upregulated the purine metabolism-related pathways of the intestinal flora and increased the abundance of KOs involved in UA degradation, suggesting that RES alters the intestinal microbiota composition in order to promote purine and UA metabolism.

Various studies have indicated that the intestinal microbiome could be a key focus for the improvement of NAFLD, diabetes, and other metabolic diseases caused by RES [60,61]. Wang et al. confirmed through FMT that RES enhanced weight loss in obese mice through restoring the gut microbiota, as indicated by the increased abundance of *Blautia* and the decreased abundance of *Desulfovibrio* and *Lachnospiraceae_NK4A13* [62]. However, whether RES degrades UA by regulating the gut flora is unknown. In this study, we employed FMT to verify the role of the gut microbiota in the ability of RES to alleviate HFD-induced HUA. In line with the findings of the intervention study, fecal bacteria from the RES group showed a significant decrease in serum and fecal UA levels, as well as an increase in UA degradation. Like the changes observed in the metagenomic analysis, the 16S rRNA analysis of the FMT experiment revealed that fecal bacteria from the RES treatment significantly enhanced the abundance of *Lactobacillales* and *Ligilactobacillus* and decreased the abundance of *Eisenbergiella*, which is reportedly associated with inflammatory disorders [63,64]. Our results suggest that RES may improve intestinal UA metabolism by reversing intestinal microbiota disturbances.

The limitation of this study is that, due to the current lack of mature technology for detecting microbial enzymes involved in UA metabolism pathways, the specific types, levels, and activities of microbial enzymes mentioned in this study need to be further verified in the future. Moreover, considering the differences in the intestinal flora between human and mouse models, additional studies, including in vitro gut fermentation and clinical trials, are needed to determine the effects of RES on the human gut microbiota and HUA. Hence, further exploration is necessary to clarify the specific underlying processes involved.

## 5. Conclusions

This study suggested that RES may reduce HFD-induced HUA and renal injury by promoting the proliferation of beneficial intestinal flora that degrade UA, improving purine metabolism-related pathways, and inhibiting harmful proinflammatory bacteria. The probiotic *Lactobacillus* may play a key role in this effect. Ultimately, this study provides an effective and safe supplemental strategy and valuable experimental evidence for the management of HUA.

## Data Availability

The data presented in this study are available on request from the corresponding author.
